# A *Dictyostelium* SH2 adaptor protein required for correct DIF-1 signaling and pattern formation

**DOI:** 10.1016/j.ydbio.2011.03.003

**Published:** 2011-05-15

**Authors:** Christopher Sugden, Susan Ross, Sarah J. Annesley, Christian Cole, Gareth Bloomfield, Alasdair Ivens, Jason Skelton, Paul R. Fisher, Geoffrey Barton, Jeffrey G. Williams

**Affiliations:** a School of Life Sciences, University of Dundee, Dow St., Dundee, DD1 5EH, UK; b Wellcome Trust Sanger Institute, Hinxton, CB10 1SA, UK; c MRC Laboratory of Molecular Biology, Hills Road, Cambridge, CB2 2QH, UK; d Department of Microbiology, La Trobe University, Bundoora, Victoria 3086, Australia

**Keywords:** *Dictyostelium*, SH2 domain, Adaptor protein, DIF-1, Prestalk, Basal disc

## Abstract

*Dictyostelium* is the only non-metazoan with functionally analyzed SH2 domains and studying them can give insights into their evolution and wider potential. LrrB has a novel domain configuration with leucine-rich repeat, 14-3-3 and SH2 protein–protein interaction modules. It is required for the correct expression of several specific genes in early development and here we characterize its role in later, multicellular development. During development in the light, slug formation in LrrB null (lrrB-) mutants is delayed relative to the parental strain, and the slugs are highly defective in phototaxis and thermotaxis. In the dark the mutant arrests development as an elongated mound, in a hitherto unreported process we term dark stalling. The developmental and phototaxis defects are cell autonomous and marker analysis shows that the pstO prestalk sub-region of the slug is aberrant in the lrrB- mutant. Expression profiling, by parallel micro-array and deep RNA sequence analyses, reveals many other alterations in prestalk-specific gene expression in lrrB- slugs, including reduced expression of the *ecmB* gene and elevated expression of *ampA*. During culmination *ampA* is ectopically expressed in the stalk, there is no expression of *ampA* and *ecmB* in the lower cup and the mutant fruiting bodies lack a basal disc. The basal disc cup derives from the pstB cells and this population is greatly reduced in the lrrB- mutant. This anatomical feature is a hallmark of mutants aberrant in signaling by DIF-1, the polyketide that induces prestalk and stalk cell differentiation. In a DIF-1 induction assay the lrrB- mutant is profoundly defective in *ecmB* activation but only marginally defective in *ecmA* induction. Thus the mutation partially uncouples these two inductive events. In early development LrrB interacts physically and functionally with CldA, another SH2 domain containing protein. However, the CldA null mutant does not phenocopy the lrrB- in its aberrant multicellular development or phototaxis defect, implying that the early and late functions of LrrB are affected in different ways. These observations, coupled with its domain structure, suggest that LrrB is an SH2 adaptor protein active in diverse developmental signaling pathways.

## Research highlights

► LrrB null (lrrB-) slugs are highly defective in phototaxis and thermotaxis, perhaps because the anterior, pstO prestalk sub-region of the slug is aberrant. ► There are many changes in gene expression in the null mutant, especially in prestalk-specific transcripts. ► The posterior, pstB sub-population of the slug is absent in the lrrB- mutant and the derivative structures are aberrant or missing. ► The lrrB- mutant is highly defective in *ecmB* induction by the polyketide DIF-1 but only marginally defective in *ecmA* induction, so the mutation partially uncouples these two inductive events. ► SH2 domain signaling in Dictyostelium mirrors that of animal cells in utilizing single adaptor proteins in multiple signaling pathways.

## Introduction

SH2 domains mediate the tyrosine phosphorylation-dependent formation of protein complexes in many cellular and developmental signaling pathways (Pawson et al., 2001). Almost all of those described are found in animals, which typically encode over a hundred SH2 domain proteins (Liu et al., 2006), but the amoebozoan *Dictyostelium* possesses a small functionally diverse contingent of thirteen SH2 domain proteins (Eichinger et al., 2005). The family includes four STAT proteins, five kinases, an ortholog of the Cbl proto-oncogene and an F-box protein. Based upon their domain structure the latter two proteins, CblA and FbxB, are assumed to target other proteins for degradation (Langenick et al., 2008). The remaining two SH2 domain proteins, CldA and LrrB, respectively contain a Clu domain and a leucine-rich repeat domain (Sugden et al., 2010).

The Clu domain is a relatively large, > 200aa, domain of unknown function. The founding family member was *Dictyostelium* CluA, a highly conserved protein that is essential for the maintenance of mitochondrial spacing (Fields et al., 2002; Fields et al., 1998; Zhu et al., 1997). Leucine-rich repeats are protein–protein interaction domains and LrrB also contains a functional binding site for 14-3-3, another regulatable protein–protein interaction site (Mackintosh, 2004). The presence in LrrB of three protein–protein interaction modules and the absence of any other recognizable domains suggest that, like CblA and FbxB, LrrB serves as an adaptor. Consistent with this, LrrB interacts with CldA and this binding depends upon an intact LrrB SH2 domain and is markedly increased when pervanadate is used to generate a high cellular level of tyrosine phosphorylation by inhibiting tyrosine dephosphorylation.

The *lrrB* gene is expressed semi-constitutively, during cell growth and throughout development, but a null mutant shows no outward defect in growth and early development (Sugden et al., 2010). However, expression-profiling, using a genome-wide microarray, revealed large (respectively 7, 44 and 49-fold) decreases in the abundance of just three mRNA sequences: encoding TtdA (a metallo-hydrolase), CinB (an esterase) and AbcG10 (an ABC transporter). There was also an increase in the abundance of the discoidin1 lectin mRNAs. The phenotypic significance of these changes and the mechanism generating them are unknown, but they were used to confirm a functional interaction between CldA and LrrB — by showing that the two genes most highly under-expressed in the lrrB- strain, cinB and abcG10, are also under-expressed in a *cldA* null strain. Thus there are very specific, but outwardly silent, defects in the early development of the lrrB- strain. Here we analyze the late phenotypes of the null mutation and show that the mutant has more frequent but less extreme aberrations in individual gene expression. There are also marked morphological defects and aberrations in slug behavior, structure and terminal development in the lrrB- mutant that seem likely to relate to defects in prestalk differentiation.

During normal development cells aggregate together and pile up to form a mound. Cells within the mound differentiate as either prestalk or prespore cells. Many of the prestalk cells move to the apex of the mound but a significant proportion, the anterior-like cells (ALC), remains in the prespore zone. The mound elongates to form a standing slug that either enters culmination immediately or falls onto its side and migrates away. The migratory slug is sensitive to light and temperature gradients and these sensitivities direct it to an appropriate microenvironment for culmination and spore release. There are multiple prestalk and ALC subtypes that can be distinguished, using molecular markers and by their anatomical positions and patterns of movement (Gaudet et al., 2008). The subtypes also differ in the extracellular signals that induce them.

DIF-1 is a chlorinated hexaphenone that is produced by the prespore cells (Kay and Thompson, 2001). It induces the expression of *ecmA* and *ecmB*, two genes that encode closely related extracellular matrix proteins. PstO cells and PstO-ALC utilize the cap-site distal region of the *ecmA* promoter and their differentiation is induced by DIF-1 (Early et al., 1993; Thompson and Kay, 2000). PstB cells express *ecmB* at a higher level than *ecmA* and their differentiation is also induced by DIF-1 (Jermyn and Williams, 1991; Keller and Thompson, 2008; Saito et al., 2008). By analyzing the development and DIF-1 induction of the lrrB- strain we derive evidence for a selective involvement of LrrB in ecmB expression.

## Materials and methods

### Cell culture, transformation, development, neutral red staining and reporter expression analysis

Ax2 cells (Gerisch isolate), and derivative lrrB- and cldA- strains, were grown axenically, transformed and developed as described previously (Sugden et al., 2010). For neutral red staining a stock solution (0.1 mg/ml) of neutral red was added to a partially resuspended vegetative cell pellet at 8 × 10^7^ cells/ml at a 100 fold final dilution in KK2 (20 mM K_2_HPO_4_/KH_2_PO_4_ pH 6.2), and the cells were mixed briefly then washed in KK2 until there was no residual color in the wash. Cell type-specific expression of lacZ reporter constructs was monitored by enzymatic staining (Dingermann et al., 1989). Generally, slugs were lifted onto and stained on glass coverslips, while all stages of culmination were directly stained on HA (Millipore) filters. GFP fluorescence of living developing structures was viewed under silicone oil (Dormann et al., 1996) by confocal microscopy.

### DIF-1 induction of monolayer cells

Exponentially growing cells were harvested, washed and plated in 6 cm plastic dishes (Nunc) at a density of 0.8 × 10^6^ cells/ml and 2 × 10^6^ cells per dish, in stalk medium (10 mM MES-KOH pH6.2, 10 mM KCl, 2 mM NaCl, 1 mM CaCl_2_). After settling cells for 1 h cAMP and cerulenin (Calbiochem) were added to final 5 mM and 50 μM concentrations respectively (N. B. the cerulenin is added to inhibit endogenous DIF-1 synthesis). After 6 h the medium was replaced with stalk medium containing 100 nM DIF-1, or control solvent (0.1% ethanol), and 50 μM cerulenin, for 16 h.

### Plasmid construction

The ecmAO-LrrBCTAP construct, expresses LrrB with a C-terminal TAP tag expressed under the entire *ecmA* promoter. It was created by removing the TAP-tagged LrrB fragment, using BamHI + XhoI*,* from LrrB-CTAP (Sugden et al., 2010.). This fragment was cloned into ecmAO:lacZ (Jermyn and Williams, 1991), after removal of the lacZ cassette, using BglII + XhoI. For CudA-tip:GFP the lacZ gene in promoter fusion construct F of Fukuzawa and Williams (2000) was removed, using BglII + XhoI*,* and replaced with GFP.

### Phototaxis and thermotaxis analyses

Where indicated phototaxis experiments used either: *Method A:* 10 μl of cells at 1 × 10^8^/ml were harvested from exponentially growing cultures, washed in KK2 buffer and spotted onto 9 cm agar plates (1.5%(w/v) agar, 0.5%(w/v) activated charcoal (Sigma C5385)) then allowed to develop under dim unidirectional light for 66 h. Slug trails were lifted from the agar surface onto transparent film and stained with 0.025%(w/v) Coomassie Brilliant Blue R in 7.5% acetic acid, 50% methanol for 5 min then excess stain was washed off in distilled water. *Method B:* Qualitative phototaxis tests were performed as described in Darcy et al. (1994). Phototaxis was scored after 48 hour incubation at 21 °C with a lateral light source. For quantitative phototaxis amoebae grown on bacteria were plated in the center of a charcoal agar plate. The phototaxis was scored as for qualitative analysis. For quantitative thermotaxis bacterially grown amoebae were plated at a density of 3 × 10^6^/cm^2^ onto the center of water agar plates and incubated for 72 h in darkness on a heat bar producing a 0.2 °C/cm gradient at the agarose surface. The arbitrary units correspond to the temperatures 14 °C at T1 and increasing in 2 °C increments to 28 °C at T8, as measured at the center of plates in separate calibration experiments. Slug trails were transferred to PVC discs, stained with Coomassie Blue and digitized. The orientation of the slug migration was analyzed using directional statistics (Fisher et al., 1981).

### Expression profiling using microarrays

RNA samples prepared from standing slugs of the parent and lrrB- strains were used to generate fluorescently labeled probes. These were hybridized with a genome-wide array containing 9247 features, and the results were analyzed, just as described previously (Sugden et al., 2010). The data can be viewed at “ArrayExpress”: accession: E-TABM-1090, Specified release date: 2011-05-31. Username: Reviewer_E-TABM-1090 Password: 1289827701517.

### Expression profiling using deep RNA sequencing

14,933,360 WT and 16,417,668 LrrB- reads were obtained from Digital Gene Expression “Illumina” sequencing performed in singlicate. Following the removal of adaptor sequences all the reads were 18 bp in length but the TopHat short read mapper requires reads of at least 20 bp (Trapnell et al., 2009). Addition of the CATG NlaIII restriction site motif to the 5′ end of all reads gave 22 bp reads allowing TopHat mapping (v1.0.13 allowing for two splice mismatches). For the WT sample 13,837,725 reads were mapped to the Ensembl Dictyostelium genome (release 55) of which 12,809,199 were uniquely mapped and 15,889,534 (14,604,093 unique) lrrB- mapped reads. Quantifying expression values with Cufflinks v0.8.2 (Trapnell et al., 2010) against the Ensembl transcriptome resulted in approximately 8500 transcripts with mapped reads for both samples, of which approximately 5200 transcripts had an FPKM value of > 5. Differential expression between WT and lrrB- transcripts was performed by comparing FPKM across the two.

### Quantitative Real-time PCR (QPCR) analysis of gene expression

Quantification of gene expression by QPCR was performed as in Sugden et al. (2010). The primers used are presented in relevant figure legends.

## Results

### Development of lrrB- cells occurs differently in darkness and in light

**Fig. 1:**
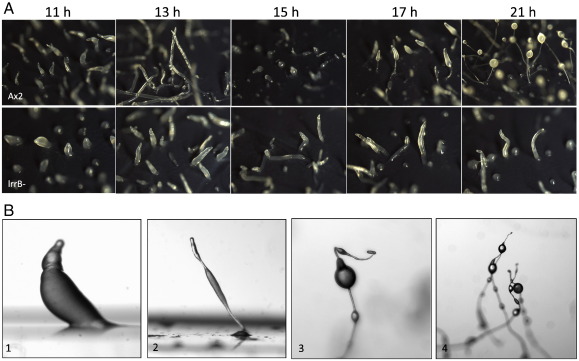
Analysis of lrrB-development. (A) Ax2 and lrrB- cells were developed on filters sitting on agar in overhead light and photographed at the stated times. (B) lrrB- cells were developed on agar in overhead light for 26 h (panels 1&2) and 43 h (panels 3&4).

**Fig. 2:**
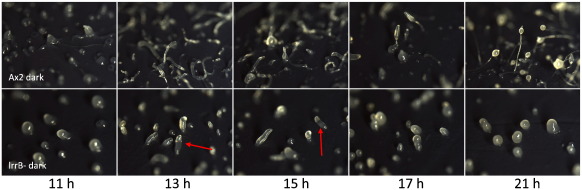
Analysis of lrrB- development in the dark. Ax2 and lrrB- cells were developed on filters sitting on agar in the dark, removed and photographed at the stated times. Arrows indicate structures noted in text.

When lrrB- cells are plated on agar plates under uniform overhead light development proceeds normally up to the tipped aggregate stage but the structures formed are relatively squat and the slug stage is markedly extended (Fig. 1A). Eventually, after 30 to 36 h, culminants are formed but these are aberrant, displaying multiple spore beadlets that are incompletely elevated up the stalk (Fig. 1B). When lrrB- cells are plated on agar in darkness development proceeds up to the tipped aggregate stage but the structures formed are squat and more apically rounded than the parental structures. They elongate, to form stumpy slug-like structures (arrowed in Fig. 2) but these rapidly transform into bullet-shaped upright mounds. These remain indefinitely arrested in their development, even when transferred into the light. We term this dark stalling, because the cells remain developmentally competent; if the arrested structures are mechanically disaggregated and transferred to the light small fruiting bodies are formed (data not shown).

### lrrB- slugs are defective in environmental responsiveness

**Fig. 3:**
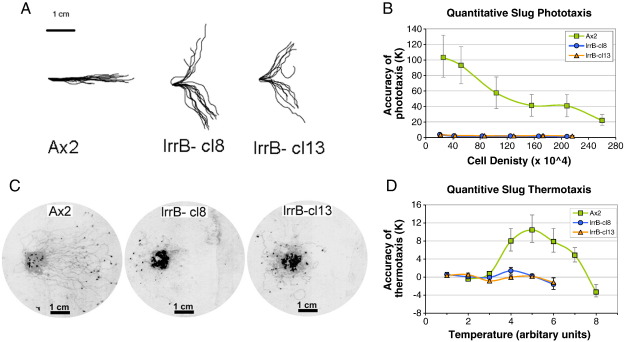
Analysis of phototaxis and thermotaxis in the lrrB- strain. (A) Qualitative slug phototaxis (method B). Digitized slug trails of wild type Ax2, and two lrrB- clones, cl8 and cl13. Slug trails were plotted from a common origin so that the source of light is to the right of the figures. Wild type slugs migrate directly toward the light source whereas lrrB- slugs show disoriented, bimodal phototaxis. (B) Quantitative phototaxis (method B) at defined cell densities was performed on wild type Ax2, and two lrrB- transformants. The trails were digitized and analyzed using directional statistics to determine their accuracies of phototaxis (κ) at the defined cell densities. In comparison to the wild type Ax2 strain the two lrrB- clones show very low accuracies of phototaxis. (C) Qualitative slug thermotaxis (method B). Scanned image of stained trails from slugs during thermotaxis in a stable, linear temperature gradient of 0.2 °C/cm. The direction toward the warmth is to the right of the figure and the temperature at the inoculation site was 20 °C (Fisher and Williams, 1982). Thermotactic orientation by the lrrb null mutants, cl8 and cl13, is strongly impaired. (D) Quantitative thermotaxis of lrrB- clones. Temperatures designated 1 through 8 correspond to temperatures from 14 °C in 2 °C steps to 28 °C, with a temperature gradient at the agar surface of 0.2 °C/cm. The slug trails were digitized and analyzed using directional statistics (Fisher et al., 1981). Positive values indicate the accuracy of positive thermotaxis (κ) toward the warmth, negative values indicate the accuracy of negative thermotaxis (κ) toward the cold and a value of zero indicates there was no preferred direction. Ax2 displayed high accuracies of positive thermotaxis at T4–T7 and low positive accuracies of thermotaxis at the cooler temperatures with Ax2 displaying negative accuracies of thermotaxis at T8. The two lrrB- clones, cl8 and cl13 displayed low accuracies of thermotaxis at all temperature points. It is normal for the accuracy of thermotaxis by wild type Ax2 slugs to decline above and below the growth temperature and either approaches zero or even switches to negative thermotaxis at T1 (Fisher et al., 1997; Darcy et al., 1994). At some of the higher temperatures slugs of the lrrB- clones did not migrate so thermotaxis could not be measured.

Wild type *Dictyostelium* slugs exhibit extremely sensitive and accurate oriented migration toward a dim lateral light source as well as highly sensitive orientation in shallow temperature gradients. These phototactic and thermotactic responses have been well studied and are known to be controlled by cells in the anterior of the slug (reviewed in Fisher, 2001), although the roles of the specific subpopulations of prestalk cells in that region are not defined. In view of the different developmental phenotypes of lrrB- cells in the light and the dark, it was of interest to determine if phototaxis and thermotaxis responses were defective. When lrrB- cells are plated on charcoal agar plates slugs are formed but they are profoundly defective in both phototaxis and thermotaxis (Fig. 3). It is well established that the signaling pathways for phototaxis and thermotaxis converge early so that most of the signaling molecules involved are required for both behaviors. The defects in phototaxis and thermotaxis by lrrB- slugs suggest either that LrrB is a direct participant in photosensory and thermosensory signaling or that it is required for the correct differentiation or localization in the anterior region of cells that control these responses.

### lrrB- slugs are defective in pstO differentiation

**Fig. 4:**
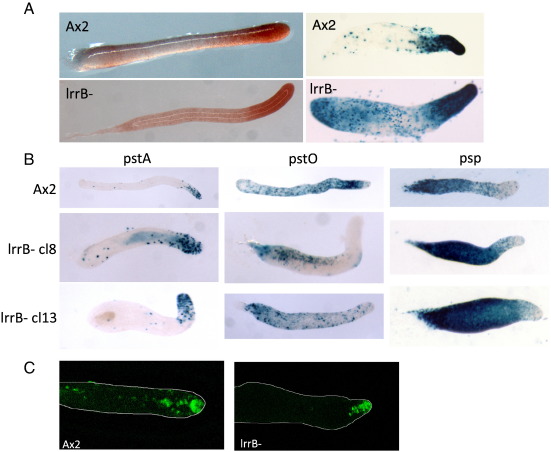
Cell type specific marker expression in Ax2 and lrrB-. (A) Ax2 and lrrB- cells were stained with neutral red, a vital dye which specifically stains prestalk cells, then developed under unidirectional light for 17 h (left panels). Ax2 and lrrB- cells, expressing the prestalk marker ecmAO:lacZ, were developed under unidirectional light, fixed and stained for *ß*-galactosidase (right panels). (B) Ax2 and 2 lrrB- clones (cl8 and cl13) expressing pstA specific, pstO specific and prespore specific markers, developed under unidirectional light, fixed and stained for *ß*-galactosidase. (C) Ax2 and lrrB- cells expressing the GFP reporter; CudA-tip:GFP, were developed under unidirectional light and GFP was visualized in the tips of the slugs. Z-stacks from confocal sections are presented with the outline of the whole slug.

To investigate the anatomy of lrrB- slugs, we used markers of prestalk and prespore differentiations. EcmAO:lacZ contains the entire promoter of the *ecmA* gene and is expressed in the pstA region, the pstO region and many of the ALC. EcmAO:lacZ expression appears normal in the mutant strain (Fig. 4A). However markers derived from the component parts of the ecmAO promoter, ecmA:lacZ and ecmO:lacZ, give a different picture — pstA differentiation appears normal but, even after extended slug migration, there is little or no ecmO:lacZ staining in the pstO zone (Fig. 4B). There is an accumulation of ecmO:lacZ stained cells at the rear of the slug, so it could, in principle, be that pstO cells are formed but fail to sort properly to the pstO region. However, we favor the alternative explanation; that there are cells at the position of the pstO band that can utilize the entire ecmAO promoter but that cannot utilize the ecmO promoter sub-region. This explanation is supported by analysis of the prespore marker pspA:lacZ, a marker of prespore differentiation (Fig. 4B), and by neutral red staining, a generic marker for prestalk cells and ALC (Fig. 4A). Both markers reveal a normal-sized prestalk zone in the lrrB- slugs.

Several key functions of the slug, such as tip dominance and the control of entry into culmination, are mediated by the tip-organizer cells; a cone of cells located in the extreme slug anterior (Gaudet et al., 2008; Rubin and Robertson, 1975; Smith and Williams, 1980). CudA is a transcription factor with a bipartite promoter. Cap-site proximal elements direct specific gene expression in prespore cells, while distal elements direct expression in tip organizer cells (Fukuzawa et al., 1997). Parental and lrrB- cells were transformed with CudA-tip:GFP, which contains a GFP reporter driven by the tip-organizer specific elements of *cudA*. The parental and mutant slugs show similar GFP expression patterns, with a cluster of fluorescent cells in the tip (Fig. 4C).

### In synergy experiment lrrB- cells are excluded from the prestalk zone

**Fig. 5:**
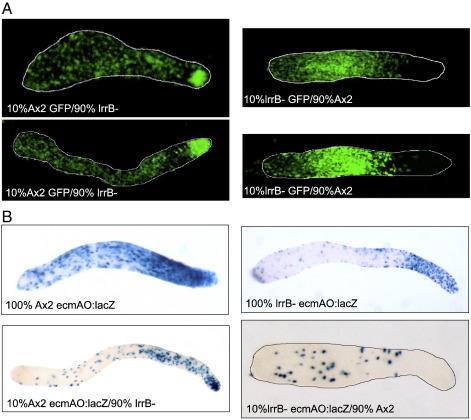
Analysis of the developmental distribution of lrrB- cells in synergy with Ax2. (A) Ax2 or lrrB- cells carrying a GFP marker under the semi-constitutive actin15 promoter (A15:GFP), were mixed in a 1:9 ratio with unlabeled cells and developed under unidirectional light for 17 h. The localization of the GFP marked cells was visualized in slugs. Z-stacks from confocal slices are presented with the outline of the whole slug. Control slugs from a synergy between cells carrying a GFP marker and the parent cells (both Ax2, and lrrB-), showed uniform prestalk/prespore distribution. (B) Ax2 or lrrB- cells expressing the prestalk marker ecmAO:lacZ, were mixed in a 1:9 ratio with the parent cells and developed under unidirectional light for 17 h. Upper panels show slugs from 100% Ax2 and lrrB- expressing ecmAO:lacZ and the lower panels the slugs from synergy experiments. Slugs are outlined where necessary.

Thus tip-organizer differentiation appears to be normal in the mutant when the mutant cells are developing alone. However, synergy experiments reveal a major defect in cell sorting behavior of the entire anterior prestalk population. Genetically marked versions of the mutant and parental strains were generated by transformation with A15:GFP, a semi-constitutively expressed GFP reporter. When an excess of unmarked lrrB- cells is co-developed to the slug stage with A15:GFP labeled parental cells, in a 9:1 mixture, the marked parental cells selectively accumulate in the front half of the prestalk zone: the pstA region (Fig. 5A). Conversely, when an excess of unlabeled parental cells are co-developed with A15:GFP marked null cells, the labeled lrrB- cells are excluded from the anterior and instead lie scattered in the rear, prespore zone: mainly concentrated toward the middle of the zone. The lack of complete reciprocity between the two arms of the synergy experiment presumably reflects a sorting hierarchy that is more complex than simple prestalk vs prespore.

A similar pattern is observed in synergy experiments using cells marked by their expression of ecmAO:lacZ as a generic marker of prestalk differentiation (Fig. 5B). Thus, in the presence of an excess of parental cells, prestalk differentiation occurs in the lrrB- mutant but the cells are excluded from the prestalk zone and concentrate toward the middle of the prespore region. This implies a cell autonomous defect in cell sorting in the null mutant. The converse experiment supports this notion, because the parental prestalk cells become enriched in the anterior of the prestalk region (Fig. 5B).

The phototaxis defect of the lrrB- strain is also cell autonomous. In a synergy experiment, using an increasing proportion of parental cells, there is a progressive improvement in the accuracy of phototaxis that plateaus at 10–25% wild type cells (data not shown). This correlates well with the results of the above experiment using GFP marked cells (Fig. 5A), which suggests that most of the prestalk cells in such a mixture are phototactically competent, parental cells.

### lrrB- slugs display aberrant gene expression patterns

The patterns of gene expression in standing slugs of parental and lrrB- strains developing under overhead light were compared by expression profiling. Profiling was performed using a microarray bearing genomic DNA fragments that represent over 80% of the predicted genes (Bloomfield et al., 2008) and a similar analysis was performed using deep RNA sequencing. For those genes that change their expression by a factor of at least two-fold there was an approximate 20% overlap between the two methods: comprising 4 genes with increased expression and 28 with decreased expression (Table 1). Strikingly, when the 28 genes with decreased expression are analyzed on dictyExpress (http://www.ailab.si/dictyexpress) for the ratio of their expression in purified prestalk and prespore cells there is a greater than three-fold imbalance. Sixteen (57%, red in Table 1) are prestalk-enriched while only five (17%, blue in Table 1) are prespore-enriched. This seems likely to be significant because of the 231 randomly selected genes, analyzed applying the same discrimination criterion, 24% were prestalk-enriched and 25% prespore-enriched (data not shown).

The results for three of the genes with increased expression and six of the genes with decreased expression (asterisked in Table 1) were confirmed by QPCR on the same RNAs used for deep RNA sequencing. Five of the genes, *ampA*, *ecmB*, *aifD*, *csaA* and *mybZ* were then re-analyzed over a mini-developmental time course encompassing the period when the lrrB- becomes visibly aberrant and these results further confirmed the differences (data not shown). Finally, full expression patterns were obtained for *ampA* and *ecmB*, by QPCR using a complete developmental time course. The *ecmB* gene is commonly used as a marker for culmination. It encodes an extracellular matrix protein that is very closely related to EcmA. AmpA is a disintegrin domain containing protein with an anti-adhesion function (Blumberg et al., 2002). For completeness, we included *ecmA* in this analysis even though its expression was not detected as being altered in the expression profiling.

Starting at about 12 h of development the lrrB- strain shows an approximately two-fold reduction in *ecmA* expression relative to the control. EcmB expression is, as expected from the microarray, deep RNA sequencing and the mini-developmental time course, > 5-fold lower than the control at later stages. AmpA is first expressed earlier in development than *ecmA* or *ecmB* and expression is initially similar in the parental and lrrB- strains. At times after 10 h of development there is a two to three fold higher level of *ampA* expression in the mutant strain. In both strains *ampA* expression decreases during culmination.

### lrrB- cells are defective in ancillary stalk cell differentiation

**Fig. 7:**
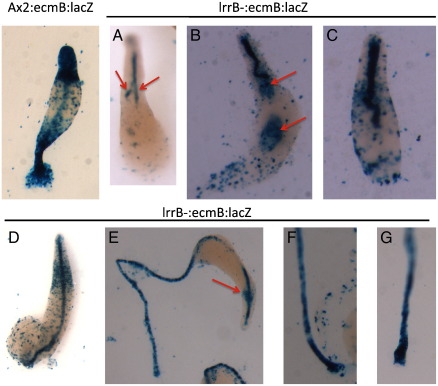
ecmB:lacZ expression in Ax2 and lrrB- culminants. Ax2 and lrrB- cells expressing ecmB:lacZ, were developed to the late culmination stage under overhead light 20 h for Ax2 and 24 h (panels A and D) and 28 h (panels B, C, E, F and G) for lrrB-, fixed and stained for *ß*-galactosidase. Arrows indicate structures noted in text.

In parental slugs ecmB:lacZ is most highly expressed in the pstAB cells: a cone of cells near the slug tip, partially over-lapping but distinct from the tip organizer cells. This is also true for the lrrB- strain but occasionally, and under conditions that we have been entirely unable to control, we also observe large circular clusters, “spots”, comprising ecmB:lacZ expressing cells, scattered throughout the prespore region (data not shown). In the lrrB- strain at culmination the stalk is much slower in its journey down through the prespore mass than that of the parental strain (Fig. 7). Sometimes there are multiple stalk fragments within one culminant (arrowed in Fig. 7A) and the stalk is generally misshapen, often forming a spiral (Figs. 7B, C and D).

Normally, two *ecmB*-expressing stalk-ancillary populations, the upper and lower cup, cradle the nascent spore head. The stalk tube becomes embedded in a third group of *ecmB*-expressing stalk cells, the outer basal disc. In the lrrB- mutant ecmB:lacZ is sporadically and weakly expressed in cells located at the approximate position normally occupied by the upper cup. These cells often form a spot very similar to those sporadically observed in the slug (arrowed in Figs. 7B and E). In the lrrB- structures there is no *ecmB* expression in the lower cup, although there are amoeboid cells at the position normally occupied by the lower cup. The outer basal disc is physically absent (Figs. 7E, F and G). In sum the above observations are consistent with the QPCR analysis, showing that the endogenous *ecmB* gene is poorly expressed in the lrrB- strain.

**Fig. 8:**
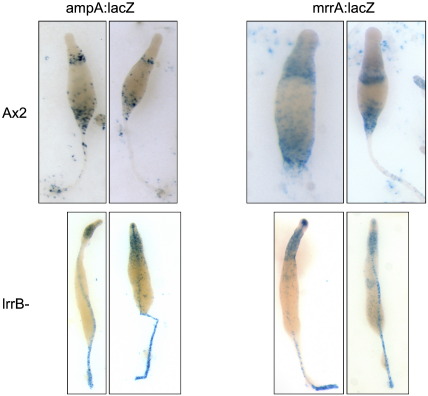
Expression of ampA:lacZ and mrrA:lacZ in Ax2 and lrrB- culminants. Ax2 and lrrB- expressing the indicated marker constructs, were developed to the late culmination stage under overhead light (17 h and 29 h respectively) then fixed and stained for *ß*-galactosidase.

We next analyzed culmination using markers specific for the stalk-ancillary structures. *AmpA* is expressed in the upper cup, lower cup and outer basal disc; expression appears to be repressed in any cells that enter the stalk tube (Casademunt et al., 2002). In parental culminants an ampA:lacZ reporter shows the expected staining pattern (Fig. 8). In lrrB- culminants ampA:lacZ is, like *ecmB*, weakly expressed in the upper cup region and it is not expressed in the cells located at the position of the lower cup. Strikingly, ampA:lacZ is also expressed, ectopically, within the stalk in lrrb- culminants. We further confirmed these observations using a reporter for *mrrA* (Tsujioka et al., 2007), another gene that is expressed in the upper and lower cups and the basal disc and that is not expressed in the stalk (Fig. 8).

**Fig. 9:**
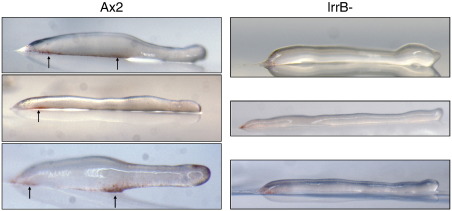
Neutral red stained cells in Ax2 and lrrB- slugs migrating on agar. The living slugs were photographed from the side. Arrows indicate the positions of pstB cells in the control Ax2 images. These position(s) are highly variable along the AP axis (Dormann et al., 1996). Note that there are also some red staining cells in the rearguard regions of all the slugs pictured. In this particular experiment the anterior prestalk regions were poorly stained (cf with Fig. 4A). Anterior staining with neutral red is intrinsically variable from experiment to experiment. However, the basal staining of pstB cells is highly reproducible and easy to visualize when slugs are viewed from the side; because they are concentrated in a very tight layer on the ventral surface.

The direct precursors of the lower cup and outer basal disc are the pstB cells, a loosely clustered group of cells that is apposed to the ventral surface of the slug. They stain strongly with neutral red and express *ecmB* at a high level relative to *ecmA* (Jermyn et al., 1996). They move continuously along the length of the slug and often accumulate near the prestalk–prespore boundary when the slug rears up (Dormann et al., 1996 and Fig. 9, lower left image). lrrB- slugs display little or no neutral red staining on the ventral surface of the slug, although there are staining cells in the extreme tail of the slug: in the rearguard (Fig. 9).

### lrrB- cells are selectively defective in DIF-1 induction of ecmB expression

**Fig. 10:**
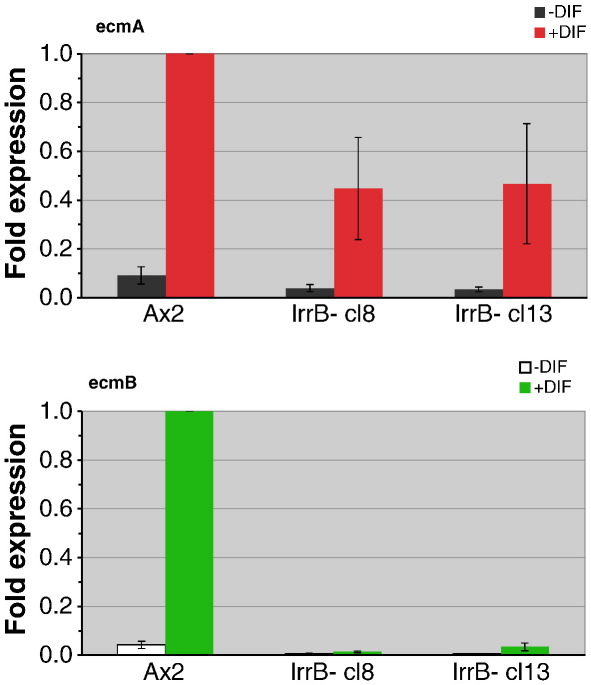
Quantitative analysis of *ecmA* and *ecmB* gene expressions. Ax2 and two lrrB-clones, cl8 and cl13, were cultured in monolayer conditions with and without DIF-1 as described in Materials and methods. RNA was prepared and analyzed for *ecmA* and *ecmB* expressions using QPCR, normalizing to Ig7 expression. Primers used were as in Fig. 6. Expression level is expressed as fold change relative to Ax2 plus DIF-1. The error bars indicate S.E.M from 4 (Ax2) and 5 (lrrB-) separate experiments.

A deficiency in lower cup and basal disc differentiation is strongly correlated with DIF-1 biosynthesis and signaling mutations (Keller and Thompson, 2008; Saito et al., 2008). Hence parental and lrrB- cells, developed in a monolayer with exogenous cAMP, were treated with DIF-1 and expression of the *ecmA* and *ecmB* genes was analyzed by QPCR (Fig. 10). Expression of the *ecmA* gene is approximately two-fold lower in the mutant strain but there is a much larger effect on *ecmB* expression, which is induced about 50-fold less efficiently in the mutant strain.

### The SH2 domain of LrrB is essential for normal development

**Fig. 11:**
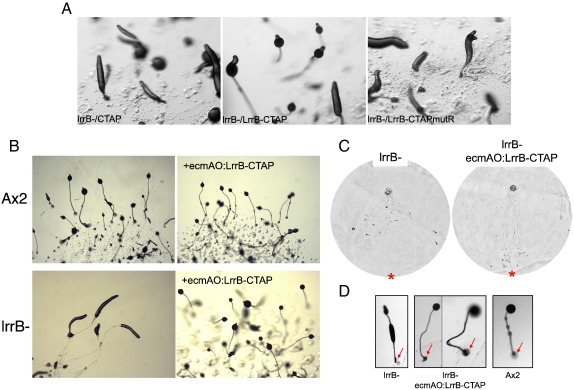
Reversion of the lrrB- developmental phenotypes by expression of lrrB. (A) lrrB- cells expressing control CTAP, LrrB-CTAP or SH2 domain point-mutated LrrB; LrrB-CTAPmutR were developed on water agar for 24 h under overhead light. The aberrant development of the lrrB- was rescued by expression of the LrrB but not by the control or SH2 domain mutated LrrB. (B) Ax2 and lrrB- cells expressing ecmAO:LrrB-CTAP were developed on water agar for 22 h under overhead light. Aberrant lrrB- culmination was rescued by expression of LrrB under the control of the prestalk specific promoter. (C) Ax2 and lrrB- cells expressing ecmAO:lrrB-CTAP were subjected to a qualitative phototaxis assay (method A). Asterisks indicate the direction of the light source. The defect in lrrB- phototaxis was rescued. (D) lrrB- cells expressing ecmAO:LrrB-CTAP and Ax2 were developed on water agar for 22 h under overhead light. Arrows indicate lack of basal disc in lrrB- and presence in ecmAO:LrrB-CTAP rescued lrrB- cells.

An essential arginine residue, present within almost all SH2 domains (arg175 in v-Src), makes critical contacts with the phosphotyrosine of its binding partner and is routinely substituted in order to inactivate SH2 domain function genetically. The early gene expression defects of lrrB- can be rescued by expression of LrrB but not by a mutant form of LrrB that lacks the essential arginine residue: arg198 in LrrB (Sugden et al., 2010). The late development phenotypes of lrrB-, prolonged and aberrant culmination, are also rescued (Fig. 11A) by expression of a TAP-tagged LrrB construct, expressed under the semi-constitutive actin15 promoter (LrrB-CTAP). Again, development is not rescued by expression of a similar construct in which arg198 is substituted by ala (LrrB-CTAPmutR).

### The late developmental defects in the lrrB- strain are reverted using an ecmAO:LrrB-CTAP construct

LrrB has effects on specific gene expression during early development and it seemed possible that these changes might exert a “knock-on” effect, indirectly causing the late developmental phenotypes. One way to investigate this possibility is to attempt to revert the late phenotypes of the lrrB- strain by expressing LrrB from a late promoter. If the effect of the mutation is indeed indirect this would allow the early defect to manifest itself late in development, i.e. late expression of LrrB should fail to rescue development of the lrrB- strain. Since it seems very likely that the overt LrrB functions in late development are exerted in prestalk cells, we used the ecmAO promoter for this purpose When stably transformed into cells the ecmAO:LrrB-CTAP construct reverts the late developmental aberrations and rescues phototaxis (Figs. 11B, C and D).

### The cldA null mutant does not display the same late developmental defects as the lrrB-

**Fig. 12:**
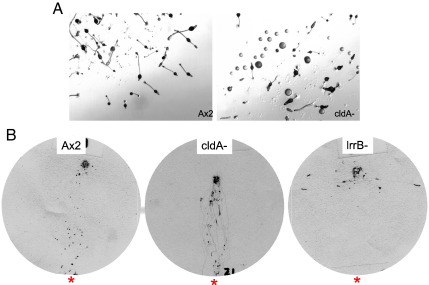
Analysis of cldA- development and phototaxis. (A) Ax2 and cldA- cells were developed on water agar for 19 h. (B) Ax2, cldA- and lrrB- cells were subjected to a qualitative phototaxis assay (method A). CldA- shows wild type phototaxis.

During early development LrrB physically interacts with CldA, a Clu domain protein that itself contains an SH2 domain (Sugden et al., 2010). The cldA- strain phenocopies the reduced early gene expression phenotype of the lrrB-. When the cldA- strain is developed on agar many of the aggregates arrest development as flattened mounds. However, a significant proportion of aggregates develop normally to form fruiting bodies with an outer basal disc (Fig. 12A). The cldA- strain also displays normal phototaxis (Fig. 12B) and does not show dark stalling (data not shown).

## Discussion

### Dark stalling

The lrrB- mutant displays a condition-dependent aberration in slug formation that is, to our knowledge, hitherto undescribed. In darkness development arrests in a process we term dark stalling. The existence of dark stalling in the mutant suggests that parental cells receive some kind of positive stimulus from the light. Since the parental cells can culminate in the absence of light the stimulatory signal is presumably redundant with a parallel, light independent system. If the lrrB- strain were defective in this parallel system this would lead to dark stalling. The fact that, even when developed in the light, development is slower in the lrrB- strain than in the parent suggests that there may be incomplete redundancy between the two proposed systems.

### Defects in slug behavior

The prestalk region of the slug controls various aspects of slug behavior, including phototaxis and thermotaxis (Fisher, 2001). Both these latter processes are highly defective in the lrrB- mutant. Although this could indicate a direct role for LrrB in photosensory and thermosensory signaling it is also possible that LrrB is required for the correct differentiation or localization of anterior cell subpopulations that are needed for phototaxis and thermotaxis. Our observation that PstO cells fail to differentiate properly in lrrB- slugs suggests that PstO cells play an essential role in slug photosensory and thermosensory responses. This is consistent with recent observations that phototaxis and thermotaxis are defective and PstO cells appear to mislocalize in a mutant lacking a novel HECT ubiquitin ligase (C.R.L. Thompson, pers. comm.). These two sets of results are the first to suggest an essential role in phototaxis and thermotaxis for a specific subpopulation of prestalk cells in the slug.

### Can defects in prestalk differentiation account for defective slug formation and behavior?

The above observations indicate aberrations in the prestalk region of lrrB- slugs. Several of the biological functions of the prestalk zone, including cAMP production, have been attributed to the tip-organizer cells. However, a reporter construct reveals normal tip-organizer regions in mutant slugs. Also ecmAO:lacZ and ecmA:lacZ, markers of prestalk differentiation, identify discrete pstO and pstA regions. Significantly perhaps ecmO:lacZ is not expressed in the pstO region of the mutant. We take this to mean that the complete promoter of the *ecmA* gene, the ecmAO region, behaves as more than the sum of its component *ecmA* and *ecmO* parts. The fact that cells in the lrrB null “pstO” region differ from parental pstO cells in not being competent to express ecmO:lacZ implies that at least one signaling pathways to gene expression differs in the mutant. Presumably, other genes are also misexpressed in the mutant pstO cells and these perhaps account for the aberrations in slug behavior.

### Other gene expression defects

Microarray analysis of gene expression shows that many genes are indeed mis-expressed in the lrrB- mutant. Expression profiling was also performed using deep RNA sequencing and we focused on those genes that show similar changes using both methods. Presumably because of the stringent selection procedure, changes in all nine genes that were re-investigated by QPCR were confirmed. As with the vegetative cells, more genes were under-expressed than were over-expressed. Significantly, given the other evidence we present for a defect in prestalk function, most of the down-regulated genes are prestalk-enriched in their expression. None of the genes is as highly mis-regulated as the vegetative/early development genes; the maximally under-expressed gene, *ecmB* is approximately 5-fold under-expressed in the standing slug while *cinB* is approximately 100-fold under-expressed at three hours of development (Sugden et al., 2010). Two genes oppositely affected by the mutation, *ecmB* and *ampA*, were studied further.

### Culmination defects

In the lrrB- strain stalk formation is very slow and the stalk is often aberrant in shape. The retarded stalk formation presumably explains why *ecmB* is so strongly under-expressed in the null strain. The stalk is also aberrant in composition. Thus *ampA* and *mrrA*, two markers normally only expressed in stalk-ancillary structures, are ectopically expressed within the stalks of lrrB- culminants. This perhaps indicates that LrrB forms part of a repressive pathway that renders the two genes inactive in cells that enter the stalk tube. The fact that *ampA* is not normally expressed in cells that enter the stalk tube also presumably explains why *ampA* expression is higher in the lrrB- strain.

The stalk-ancillary structures are also aberrant in the mutant. There is no basal disc and *ecmB*, *ampA* and *mrrA* are not expressed in the lower cup. The precursor population of the lower cup and basal disc, the pstB cell cluster, is as might be predicted much reduced or absent. All three genes analyzed show weak expression in cells in the approximate position of the upper cup. Interestingly, expression of *mrrA* and of *ecmB* in the lower cup is also abolished in a null strain for MybE, a Myb family transcription factor (Tsujioka et al., 2007), and MybZ is one of the genes under-expressed in the lrrB- strain. Elevation of the spore head up the stalk is powered by a lifting movement predominantly exerted by the upper cup cells (Mujumdar et al., 2009; Sternfeld, 1998) but apparently also requiring lower cup cells (Tsujioka et al., 2007; Saito et al., 2008); so a change in their differentiation state could help explain the incomplete spore-head ascension that is frequently observed in the lrrB- strain.

### DIF-1 signaling

Mutants in DIF-1 biosynthesis and DIF-1 signaling differentiate to form culminants that lack an outer basal disc and this is also true for the lrrB- strains. The DIF-1 signaling pathway that directs pstO-specific gene expression is partly understood. DimA and DimB, two b-ZIP transcription factors, and MybE are involved (Fukuzawa et al., 2006; Huang et al., 2006; Thompson et al., 2004; Zhukovskaya et al., 2006). Although there is an apparent defect in pstO differentiation in the lrrB- strain, the DIF-1 induced *ecmA* gene is only two fold less well induced than in control cells. However, there is an almost complete ablation of DIF-1-induced *ecmB* gene expression in the mutant. Thus *ecmA* and *ecmB* can be at least partially uncoupled in their induction by DIF-1 in monolayer cells.

There is other, prior evidence for a divergence of these two signaling pathways. Under the induction conditions used in their original identification *ecmA* responded by an increase in the rate of its mRNA accumulation just 15 min after DIF-1 addition while *ecmB* responded only after several hours (Jermyn et al., 1987). EcmA induction by DIF-1 is stimulated by the simultaneous addition of cAMP while *ecmB* induction is repressed (Berks and Kay, 1990). There are, however, some clear mechanistic links. Analyses of null strains show that DimA, DimB and MybE are all required for *ecmB* induction by DIF-1 (Fukuzawa et al., 2006; Huang et al., 2006; Thompson et al., 2004; Zhukovskaya et al., 2006). Although there is as yet no evidence for any of these factors having a direct interaction with the *ecmB* promoter, the *mrrA* promoter is known to contain an essential Myb site (Tsujioka et al., 2007). The lrrB- strain provides a potential starting point to further dissect the induction pathway.

### Functional analyses of LrrB and its interaction partner

The lrrB- strain displays multiple defects in multicellular development and slug behavior but these could, in principle, all be the consequence of an earlier dysfunction. This possibility is rendered less likely by our demonstration that the late phenotypes of the lrrB- strain can be reverted by expressing LrrB under the control of a prestalk-specific promoter. The fact that a prestalk promoter can act in this way also supports the notion that the primary lesion(s) are indeed in prestalk cell function.

LrrB contains, in addition to its SH2 domain, two protein–protein interaction modules: leucine-rich repeats and 14-3-3 binding sites. It is therefore relevant that we could also show, by a mutant rescue experiment using a semi-constitutive promoter, that the SH2 domain continues to be essential at late stages. Interaction of LrrB with CldA in early development depends upon an intact LrrB SH2 domain and elevated tyrosine phosphorylation levels, generated by the addition of pervanadate to the cells (Sugden et al., 2010). One potential, but by no means exclusive, explanation of these results is that the LrrB SH2 domain binds a regulatable phosphotyrosine residue on CldA. It would seem probable, from analysis of the development and phototaxis of a cldA- strain, that LrrB utilizes a different interaction partner during the later stages of development. The fact that LrrB has such diverse roles in late development makes it probable that, as with metazoan SH2 adaptor proteins such as GRB2 (Cheng et al., 1998; Felsch et al., 1998; Hardy et al., 2007; Mainiero et al., 1995; Nizzari et al., 2007; Scaplehorn et al., 2002; Skolnik et al., 1993; Wang et al., 1995; Zhang et al., 2003), LrrB interacts with more than one effector protein to participate in multiple signaling pathways.

## Figures and Tables

**Fig. 6:**
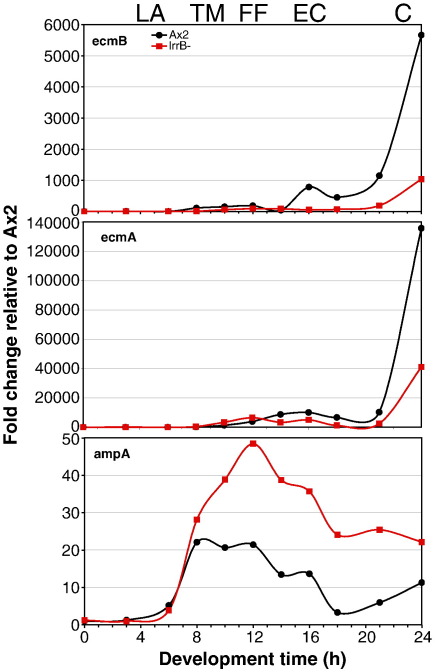
Developmental time course of the expression of *ecmB*, *ecmA* and *ampA*. Ax2 and lrrB- cells were developed on nitrocellulose filters on agar under uniform light conditions. RNA was prepared from samples harvested at the times indicated and analyzed for *ecmA*, *ecmB* and *ampA* expression using QPCR. Expression level is expressed as fold change relative to vegetative Ax2, normalizing to Ig7 expression. Developmental stage marks indicate the approximate midpoint of developmental stage in Ax2. LA, loose aggregate; TM, tight mound; FF, first finger; EC, early culminant and C, culminant. Primers used were as follows for *ecmA* CCGTAAACTGTGAATGTGATGACC (forward) and GTCTTGGAATCGCAACTATCAGC (reverse); for *ecmB* CTCTTGATTCATGTTGTTCAACTGG (forward) and CATCGCCACATTTTCCAAATG (reverse) and for *ampA* TGACCGATTCTTGTTGTGGTCGTTG (forward) and TTTGGTTGGTGGGAGAGTACATGGAC (reverse).

**Table 1:**
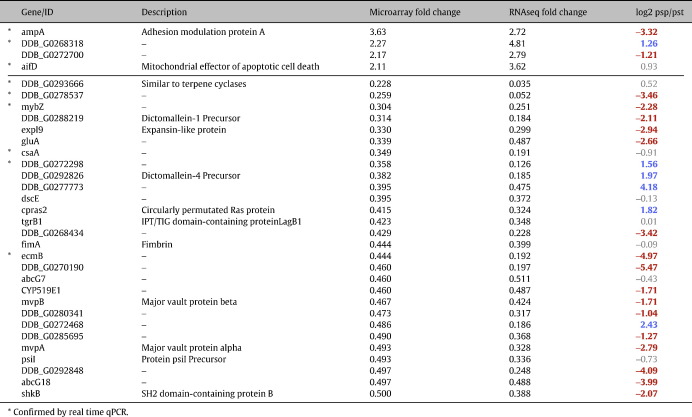
Gene-expression profiling in Ax2 and lrrB- standing slugs. Genes that showed a twofold change or more in the lrrB- from both RNA sequencing and microarray analysis are listed. Asterisks mark the changes which have been confirmed by QPCR (data not shown). The prespore or prestalk enrichment of each gene is presented as log2 ratio and was obtained from dictyExpress (http://www.ailab.si/dictyexpress). Negative values in red indicate a more than twofold prestalk enrichment, and positive values in blue indicate a more than twofold prespore enrichment.
